# It’s All Due to the Thyroid: Lessons Learnt From a Patient's Perspective

**DOI:** 10.7759/cureus.15876

**Published:** 2021-06-23

**Authors:** Kira Schaab, Thoyaja Koritala, Ramesh Adhikari, Romil Singh, Vishwanath Pattan

**Affiliations:** 1 Family Medicine, The University of Wyoming Family Medicine Residency Program, Casper, USA; 2 Internal Medicine/Hospital Medicine, Mayo Clinic Health System, Mankato, USA; 3 Hospital Medicine, Franciscan Health, Lafayette, USA; 4 Geriatrics, Brown University, Providence, USA; 5 Critical Care, Mayo Clinic, Rochester, USA; 6 Endocrinology, Wyoming Medical Center, Casper, USA

**Keywords:** thyroid, hashimoto's hypothyroidism, dermatomyositis, fatigue, dysphagia, proximal muscle weakness, health literacy

## Abstract

Symptoms associated with thyroid pathology can mimic and overlap with a myriad of other diagnostic possibilities. Based on the patient's educational status, underlying fear, anxiety, online medical search, the patient can erroneously attribute various symptoms to thyroid pathology.

We present a case of a 79-year-old female with a history of Hashimoto's hypothyroidism, meningioma, who erroneously attributed many of her symptoms to hypothyroidism despite having normal thyroid labs. The patient had symptoms of fatigue, dysphagia, and proximal muscle weakness. Surprisingly the patient already had an existing diagnosis of dermatomyositis and Zenker's diverticulum which could clearly explain her above symptoms. Moreover, the patient did not follow up for whole body scan and other tests that were ordered for cancer screening, which is the standard practice for dermatomyositis.

The patient helped us identify the deficiencies in the current health system regarding patient counseling. We identified factors that could act as communication barriers if not properly addressed which include: (1) patient’s prior medical knowledge, (2) patient’s own underlying fears about their health conditions, (3) use of effective patient education tools, (4) minimizing or avoiding use of medical jargon, (5) role switching to verify patient's understanding, (6) repetition of relevant information, and (7) involvement of the patient in shared decision making.

It is important to recognize that thyroid gland dysfunction is the most commonly self-diagnosed condition by patients and the blame can be shifted to thyroid despite evidence to the contrary if effective patient education and counseling are lacking. Understanding the psychological state of the patient along with addressing the underlying fears, and effective patient education with repetition is the key for patient compliance and management.

## Introduction

Hypothyroidism is a very common thyroid pathology characterized by thyroid hormone deficiency with prevalence rates of 3%-7% in the United States [1]. The most common cause of hypothyroidism is primary hypothyroidism due to autoimmune thyroiditis leading to thyroid hormone deficiency. Less common causes of hypothyroidism are secondary hypothyroidism due to pituitary pathology (thyroid stimulating hormone, TSH deficiency) or tertiary hypothyroidism due to hypothalamic pathology (thyrotrophin-releasing hormone deficiency). The majority of patients are either asymptomatic or have mild non-specific symptoms, but rarely the clinical presentation can potentially be fatal with severe hypothyroidism and myxedema coma. Elderly patients are more likely to present with non-specific symptoms.

Therefore, thyroid pathology can present itself in many ways, and symptoms often overlap with those seen in other conditions. Thyroid conditions are commonly discussed online and in the community, and it is easy to attribute symptoms to frequently discussed conditions that the patient could relate to, especially the thyroid. As healthcare providers, it is our responsibility to effectively relay appropriate information to our patients about their health conditions, a task that may be more difficult, especially when a health condition is less common. In this case report, we discuss the challenges associated with patient perceptions and practice trends of healthcare providers that may pose barriers to effective communication and patient care.

## Case presentation

A 79-year-old female with a past medical history of meningioma presented with the chief complaint of “it’s all due to the thyroid.” The patient was experiencing fatigue, dysphagia, and proximal muscle weakness, which she attributed to her thyroid. She had a history of Hashimoto’s hypothyroidism since the age of 40 and was experiencing heightened anxiety about her thyroid recently. One daughter recently had a thyroidectomy and her other daughter had ongoing “uncontrolled thyroid problems.” She had been in good health until 2017 when she had started to develop facial swelling and a maculopapular rash on her neck and back. She had initially presented to her primary care provider with these symptoms, which responded well to a short course of prednisone. The patient’s symptoms had eventually reappeared more aggressively in the form of a maculopapular rash on the face and neck, which had prompted her to see a dermatologist in August 2018. She was treated with prednisone and a provisional diagnosis of dermatomyositis was made.

In November of 2018, the patient had developed Gottron papules, progressively worsening dysphagia, speech difficulties, and proximal muscle weakness, so her primary care provider had referred the patient to a neurologist and an ear nose throat specialist. In the next month, a barium swallow was done which showed a 1 cm x 1.3 cm x 4 mm Zenker’s diverticulum. She lost more than 20 lbs due to dysphagia. Finally, in January 2019, the patient had been definitively diagnosed with dermatomyositis based on the presence of Gottron papules and the result of a punch biopsy taken from her right dorsal metacarpophalangeal joint that showed perivascular lymphocytic infiltrate. A follow-up test to look for malignancy such as a full-body scan had been ordered, but the patient never followed up with them.

On examination, pulse rate was 60/min (regular), blood pressure (BP) was 124/62 mmHg, respiration was 16 breaths/min, and oxygen saturation was 96% on room air. The thyroid gland was not palpable. She had 3/5 muscle strength bilaterally in the upper and lower extremities. No rash was noted. The rest of the physical exam was unremarkable.

We revisited the patient’s disease course. The patient was on levothyroxine 50 mcg daily and thyroid labs (Table 1) showed TSH to be 1.83 mIU/L (reference range 0.44-4.5 mIU/L) and free thyroxine to be 1.48 ng/dL (reference range 0.82-1.77 ng/dL).

**Table 1:** Thyroid labs. TSH, thyroid stimulating hormone; T4, thyroxine or tetraiodothyronine

Test name	Lab value	Reference range
TSH	1.83 mIU/L	0.44-4.5 mIU/L
Free T4	1.48 ng/dL	0.82-1.77 ng/dL

The patient explained that she had been taking prednisone for her dermatitis and was currently concerned about her muscle weakness and dysphagia. The patient attributed her symptoms of dysphagia, proximal muscle weakness, weight loss, and skin rash to Hashimoto's hypothyroidism. She came to us to confirm her suspicions about her thyroid. She was unaware of the symptomatic features of dermatomyositis and did not understand the increased risk of malignancy associated with the disease [2]. 

We were alarmed by her lack of insight for cancer surveillance, and by the fact that she had continued to search for an explanation for her dysphagia and proximal muscle weakness, despite having been diagnosed with dermatomyositis 10 months prior. Moreover, she also was diagnosed with Zenker’s diverticulum. We asked her how she learns new information best, and she explained how she likes to “understand the entire picture.” She liked it when information was split up into smaller concepts and when pictures were used to explain the pathology. She could not understand the medical terminologies used to explain to her about the diagnoses and pathology. She also felt that repetition helped her retain the information. We explained to her the disease pathophysiology and disease course using photo-aids and images. We switched roles and had the patient teach us back the pathology and explanation of her symptoms. We then repeated some of the facts about disease course and cancer surveillance. She felt more confident when explanations did not involve medical jargon. The patient eventually underwent recommended scans (whole body scan and CT scan) and there was no evidence of malignancy.

## Discussion

The patient’s impression of her diagnosis was that she was taking prednisone for her “dermatitis” and that her thyroid may have been causing her skin rash, dysphagia, weight loss, and proximal muscle weakness. She was unaware of the symptomatic features of dermatomyositis and did not understand the increased risk of malignancy associated with the disease. It was surprising because the patient already had an existing diagnosis of dermatomyositis and Zenker's diverticulum which could clearly explain her above symptoms [3-4]. Moreover, the patient did not follow up for a whole body scan and other tests that were ordered for cancer screening, which is the standard practice for dermatomyositis [3]. Although musculoskeletal symptoms like muscle weakness, muscle aches, stiffness are common in hypothyroidism, these symptoms are unlikely to be due to hypothyroidism, if the patient is adequately replaced with levothyroxine to achieve normal TSH as seen in our patient. Hashimoto’s thyroiditis can be attributed to difficulty swallowing, only if the size of the thyroid gland was significantly enlarged leading to compressive dysphagia. The thyroid gland was not palpable in our patient and is unlikely to be the cause of dysphagia.

This case leads us to make the following observations noted in Table 2: 

**Table 2:** Take home messages.

Observations
The patient had seen multiple healthcare providers and had looked up information on Google, which resulted in several differential diagnoses. She was confused and may have found it easier to attribute her symptoms to a more familiar condition.
It is essential to address the underlying fears of our patients. Her heightened anxiety about her thyroid could have caused her to disregard the information that she had learned about dermatomyositis.
We should minimize the use of medical jargon. Explaining the disease course in simple language that the patient can understand is very important [5].
Explanations using photo aids were well received by the patient.
After educating the patient about their new diagnosis, an effective teaching method may be to switch roles and ask the patient questions on their diagnosis to ensure proper insight into their diagnosis, disease course, and management steps.
Repetition of information was very helpful for the patient.
Involving patients in shared decision making, promotes patient trust and compliance [6].

The patient helped us identify the deficiencies in the current health system regarding patient counseling. There is a trend of healthcare providers spending more time with electronic charting, documentation, and less time for face-to-face patient counseling. There is also an increasing trend of patients spending more time online for self-diagnosis. These trends are concerning because they lead to communication barriers and can adversely affect patient outcomes. It is our responsibility to clarify information for our patients. Understanding how best to transfer information to our patients may improve patient understanding of their disease, increase compliance to medication and follow-up tests, and decrease anxiety. If patients fail to comprehend despite addressing the observations made in Table 2, then evaluation for memory impairment would be reasonable.

## Conclusions

It is important to recognize that thyroid gland dysfunction is the most commonly self-diagnosed condition by patients and the blame can be shifted to thyroid, despite evidence to the contrary, if effective patient education and counseling are lacking. An understanding of the psychological state of the patient, addressing the underlying fears, and an effective patient education with repetition is the key for patient compliance and management.
